# Adenosine Kinase couples sensing of cellular potassium depletion to purine metabolism

**DOI:** 10.1038/s41598-018-30418-5

**Published:** 2018-08-10

**Authors:** Renata Rocha de Oliveira, Raphael Morales-Neto, Silvana Aparecida Rocco, Maurício Luis Sforça, Carla Cristina Polo, Celisa Caldana Costa Tonoli, Gustavo Fernando Mercaldi, Artur Torres Cordeiro, Mário Tyago Murakami, Kleber Gomes Franchini

**Affiliations:** 1 Brazilian Biosciences National Laboratory, Brazilian Center for Research in Energy and Materials, Campinas, São Paulo 13083-970 Brazil; 2 Department of Internal Medicine, School of Medicine, University of Campinas, Campinas, São Paulo 13081-970 Brazil

## Abstract

Adenosine Kinase (ADK) regulates the cellular levels of adenosine (ADO) by fine-tuning its metabolic clearance. The transfer of γ-phosphate from ATP to ADO by ADK involves regulation by the substrates and products, as well as by Mg^2+^ and inorganic phosphate. Here we present new crystal structures of mouse ADK (mADK) binary (mADK:ADO; 1.2 Å) and ternary (mADK:ADO:ADP; 1.8 Å) complexes. In accordance with the structural demonstration of ADO occupancy of the ATP binding site, kinetic studies confirmed a competitive model of auto-inhibition of ADK by ADO. In the ternary complex, a K^+^ ion is hexacoordinated between loops adjacent to the ATP binding site, where Asp310 connects the K^+^ coordination sphere to the ATP binding site through an anion hole structure. Nuclear Magnetic Resonance 2D ^15^N-^1^H HSQC experiments revealed that the binding of K^+^ perturbs Asp310 and residues of adjacent helices 14 and 15, engaging a transition to a catalytically productive structure. Consistent with the structural data, the mutants D310A and D310P are catalytically deficient and loose responsiveness to K^+^. Saturation Transfer Difference spectra of ATPγS provided evidence for an unfavorable interaction of the mADK D310P mutant for ATP. Reductions in K^+^ concentration diminish, whereas increases enhance the *in vitro* activity of mADK (maximum of 2.5-fold; apparent *K*_d_ = 10.4 mM). Mechanistically, K^+^ increases the catalytic turnover (*K*_cat_) but does not affect the affinity of mADK for ADO or ATP. Depletion of intracellular K^+^ inhibited, while its restoration was accompanied by a full recovery of cellular ADK activity. Together, this novel dataset reveals the molecular basis of the allosteric activation of ADK by K^+^ and highlights the role of ADK in connecting depletion of intracellular K^+^ to the regulation of purine metabolism.

## Introduction

Adenosine Kinase (ADK; EC 2.7.1.20) catalyzes the phosphorylation of adenosine (ADO) to 5′-AMP using ATP as a source of phosphate^1^. This reaction is a committed step in the purine nucleotide salvage pathway, involved in maintaining the proper levels of cellular nucleotides^2^. In fact, phosphorylation by ADK keeps the intracellular levels of ADO from building up and maintains an out-in concentration gradient across the cell membrane, which makes the cytosol a sink for extracellular ADO under physiological conditions^3^. Accordingly, changes in the ADK catalytic activity translate into significant changes in intra- and extracellular ADO. For instance, hypoxia-induced inhibition of ADK potentiates intracellular accumulation and the release of ADO to the extracellular milieu^4^. Similarly, the pharmacological inhibition of ADK increases the levels of ADO and evokes a broad range of adenosine-mediated cell signaling^5,6^. Also, ADK deficiency caused by loss-of-function mutations leads to the accumulation of ADO in organs such as brain and liver^7,8^. Although many studies have previously focused on the structure and function of ADK, the mechanisms that regulate the catalytic activity of ADK are not yet fully understood.

Several factors have been shown to affect the activity of ADK. ADO itself is a primary regulator of ADK, as increased levels of intracellular ADO inhibit the enzyme’s catalytic activity through a yet unclear mechanism^9^. In this respect, increases in ADO concentration from the nanomolar to the micromolar range are needed to substantially reduce ADK activity, suggesting that this mechanism is unlikely to influence ADK activity before extreme increases of intracellular ADO are reached^10^. Besides, ADP, AMP, and Pi in excess, as well as the lack of ATP may all inhibit ADK to some extent^11–14^. ADK activity increases as the levels of free Mg^2+^ in the reaction increase up to an optimal concentration, after which point additional free Mg^2+^ leads to inhibition of the enzyme^15^.

In this study, we determined the crystal structure of mouse ADK (mADK) as a binary complex with ADO (mADK:ADO) bound to the nucleotide-binding site, and as a ternary complex with ADO occupying the high-affinity nucleoside site and ADP the nucleotide-binding site (mADK:ADO:ADP). We demonstrate, for the first time, the presence of a K^+^ in the large domain structure of mADK, adjacent to the ATP binding site. Kinetic measurements and mutational data indicate that K^+^ influences enzyme activity in such a way that K^+^ ion depletion is accompanied by inhibition of ADK. The observation that fluctuations of intracellular K^+^ parallel changes in ADK activity further supports this conclusion. Overall, these data motivate us to propose that functionally, inhibition of ADK by low levels of K^+^ may serve to regulate the intracellular levels of ADO and other purine metabolites under pathophysiological situations that are accompanied by depletion of the K^+^ ion such as ischemia and hypoxia, during which purine metabolites may play a major protective role against cellular injury.

## Results

### General features of the mADK structures

**Figure 1:**
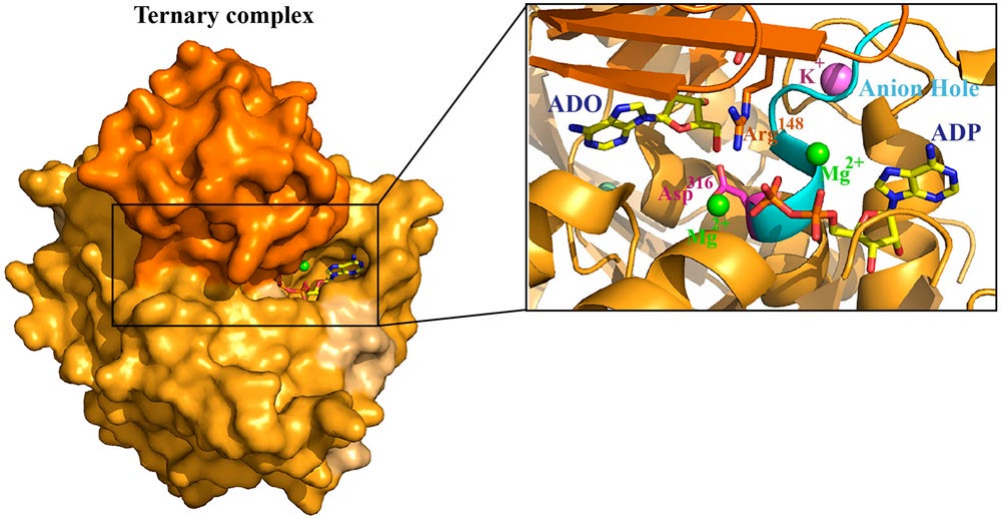
Crystal structure of mADK ternary complex highlighting the catalytic pocket, bound ADP (BS1) and ADO (BS2) and functionally relevant ions. The structure is represented in surface, highlighting the lid domain in dark orange and the large domain in light orange colors. ADP and ADO are represented in yellow sticks with carbon, oxygen, nitrogen and phosphorus atoms colored as yellow, red, blue and orange, respectively. Arg148 (orange stick), Asp316 catalytic residue (pink stick), the anion hole motif DTNGAG (cyan region), ADO in the BS2, ADP in the BS1, two magnesium ions (green spheres), one chloride ion (palecyan sphere) and one potassium ion (purple sphere).

Crystal structures of Δ19 mADK complexed with adenosine (mADK:ADO) and full- length-mADK complexed with adenosine and adenosine-5′-diphosphate (mADK:ADO:ADP) were determined at 1.2 and 1.8 Å resolution, respectively (Table 1). The final models include the residues 19 to 361 of mADK:ADO (Supplementary Fig. 1a) and 20 to 360 of mADK:ADO:ADP (Supplementary Fig. 1b). Crystals from mADK:ADO grew in the monoclinic space group P1,2_1_,1, while crystals from mADK:ADO:ADP grew in the orthorhombic space group P2_1_,2_1_,2_1_. The enzyme consists of two unequally sized domains (i.e., a large three-layer (αβα) sandwich domain and a small lid α/β domain), closely matched to the previously reported human ADK^16^ (Supplementary Fig. 2a) The large domain possesses two binding cavities that are the sites for ATP (BS1) and ADO (BS2). The structures shown here feature the BS1 and BS2 binding sites, the catalytic base Asp316 and an anion hole motif (DTNGAG) in the large domain, whereas the small or lid domain contains the residue Arg148 that is involved in binding to ATP (Fig. 1). In addition to the substrates of each complex, we observed functionally relevant ions in the ternary complex including one potassium, one chloride and two magnesium ions.

**Table 1:** Data collection and refinement statistics.

	ADO complex	ADO:ADP complex
Wavelength (Å)	1.458	1.458
Resolution range	20.13–1.20 (1.24–1.20)*	24.63–1.80 (1.86–1.80)
Space group	P 1 2_1_ 1	P 2_1_ 2_1_ 2_1_
Unit cell parameters (Å, °)	50.84, 82.16, 89.19 90, 92.7, 90	49.26 73.56 84.33 90, 90, 90
Unique reflections	227,981 (22,546)	28,498 (2,773)
Multiplicity	4.0 (3.8)	4.2 (4.1)
Completeness (%)	99.9 (99.8)	98.8 (97.7)
Mean I/sigma(I)	22.9 (2.02)	12.8 (2.04)
R-meas	0.054 (0.653)	0.058 (0.609)
Reflections used in refinement	216,557 (22,527)	27,048 (2,773)
Reflections used for R-free	11,429 (1,099)	1,450 (138)
R-work	0.141 (0.274)	0.165 (0.237)
R-free	0.159 (0.299)	0.199 (0.258)
Protein residues	686 (2 chains)	341 (1 chain)
Ligands	2 ADO, 2 CL, 3 PG4, 2 SO4 and 2 ACT	1 ADO, 1 ADP, 1 K, 1 CL, 2 MG, 2 PG4 and 2 PO4
RMS(bonds)	0.010	0.018
RMS(angles)	1.38	1.85
Ramachandran favored (%)	97.6	97.4
Ramachandran allowed (%)	2.4	2.6
Ramachandran outliers (%)	0	0
Rotamer outliers (%)	0.52	0.35
Clashscore	1.90	4.74
Average B-factor	20.01	24.21
Macromolecules	19.44	24.16
Ligands	21.94	28.55
Solvent	31.54	30.50

^*^Statistics for the highest-resolution shell are shown in parentheses.

### Interactions involved in ADO and ADP binding

**Figure 2:**
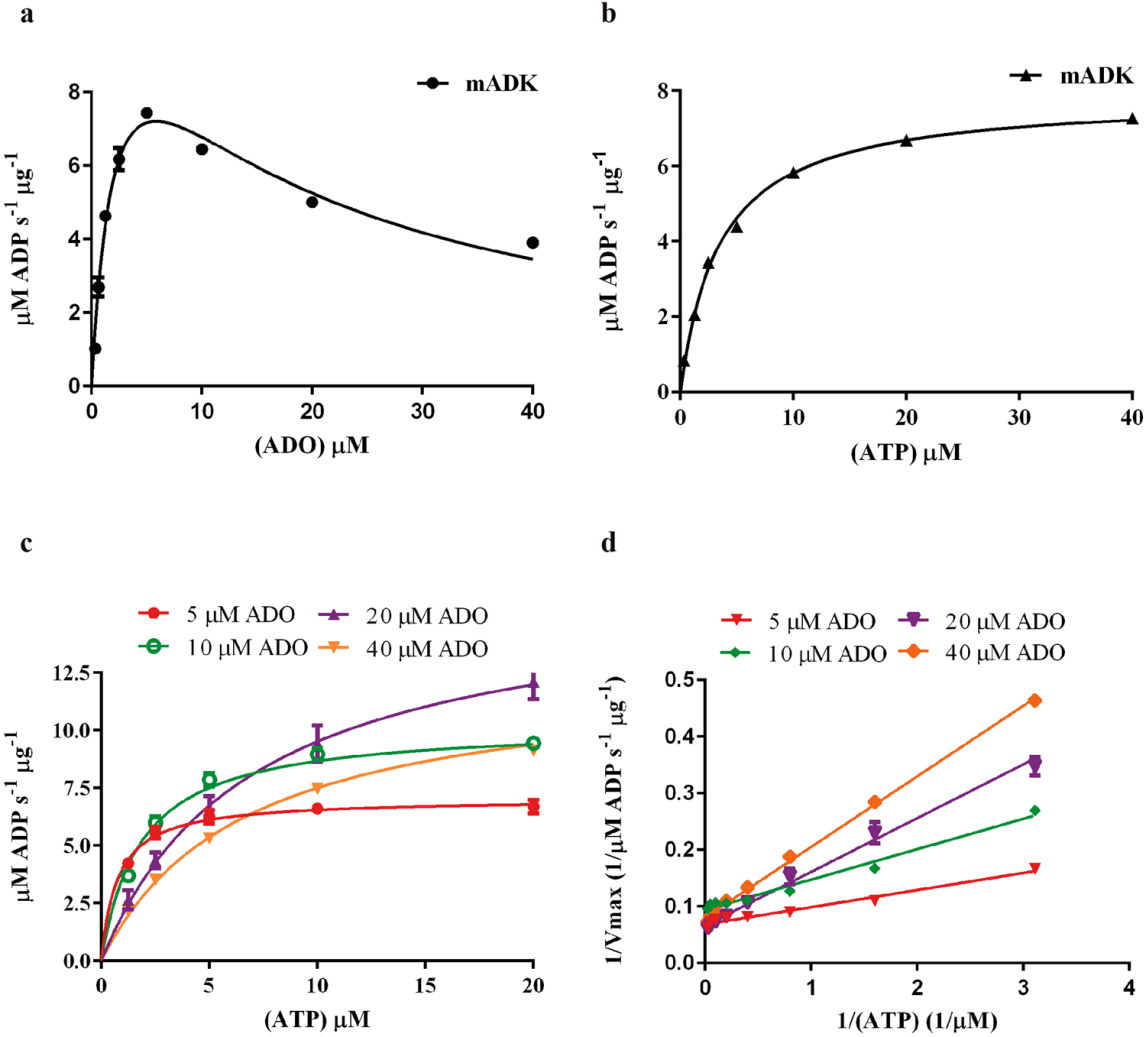
Determination of mADK kinetic parameters and substrate inhibition study. The mADK kinase activity was monitored through resorufin fluorescence which is directly related to the amount of ADP converted to the ADHP (10-acetyl-3,7- dihydroxyphenoxazine) dye precursor. The signals were measured at 530 nm (excitation) and 590 nm (emission) wavelengths. (**a**) Apparent *K*m curve for ADO, employing 15 mM mADK, 20 µM ATP and ADO from 0 to 40 µM. ADO concentrations above 5 μM inhibited the mADK. (**b**) Km curve for ATP, employing 15 mM mADK, 10 µM ADO and ATP from 0 to 40 µM. mADK presented characteristic Michaelis Menten Kinetic for the ATP substrate. Constants for ADO and ATP are shown in Supplementary Table 1. **(c)** *Substrate competition* studies evaluated by ATP *K*m curves over a range of ADO concentrations (5, 10, 20 and 40 µM). Stepwise increases of ADO reduce the affinity of mADK to ATP, as indicated by the parallel increases of the Km (Supplementary Table 2). **(d)** The mechanism of ADO inhibition was assessed by double reciprocal Lineweaver-Burk plots of 1/*v* versus 1/[ATP] µM data, which revealed that ADO is a competitive inhibitor of ATP. Reactions were carried out at 303 K.

In the mADK:ADO complex, electronic density for ADO was identified in the BS1 site, which is a large hydrophobic cluster involving residues Ile308, Val300, Ala314, Phe318, Ala343, Ile347 and polar residues Thr281, Arg284, Asp302, Gln305, His340, Gln303, Thr287, Thr311 and Gln282 (Supplementary Fig. 3a). In this site, ADO assumes a standard anti-conformation with a glycosidic torsion angle of −165.4°, while the sugar ring adopts the common C3′-endo conformation. The adenine group is hydrogen bonded to Gln305, involving atoms N1-NE2 and N6-OE1. The ribose ring makes no direct contact with the enzyme but instead establishes water-mediated interactions with the main chain atoms of residues Thr281, Gly283, and His340. Considering the peculiar occupancy of BS1 by ADO in both mADK (PDB: 5KB6) and HsADK (PDB:1BX4) binary complexes, and the potential importance of such an occupancy to the mechanism of auto-inhibition of ADK by the substrate, we next performed a detailed analysis of the superposed structures of both mADK and HsADK binary complexes (Supplementary Fig. 2a). The superposition of these binary complexes showed marked differences between the structures (overall r.m.s.d. 3.14 Å, 9.74 Å to the lid and 0.77 Å to the large domain). Distinct from the mADK, the HsADK binary structure assumes a closed conformation (~34.6° bending of the lid over the large domain), which agrees with the notion^17,18^ that the occupancy of the BS2 site by the substrate is a major factor in determining the closure of the lid over the large domain in the active form of the enzyme. Moreover, a detailed analysis of the BS1 in the superposed structures of mADK and HsADK binary complexes showed that ADO occupies a similar pocket of BS1 in both structures (Supplementary Fig. 2a). This configuration suggests that ADO may compete with ATP for the binding to BS1, which could impact on the auto-inhibitory influence of ADO on ADK. Thus, we next sought to explore the potential mechanism of ADO auto-inhibitory influence on ADK. The inhibition of ADK by ADO and the Michaelis-Menten kinetics for ATP are shown in the Fig. 2a,b, and Supplementary Table 1. In addition, we also show that stepwise increases of ADO reduce the affinity of mADK to ATP, as indicated by the parallel increases of the *K*_m_ (Fig. 2c). The mechanism of ADO inhibition was assessed by double reciprocal Lineweaver-Burk plots data (Fig. 2d). The apparent *K*_m_ values observed for the ATP are higher, the greater the concentrations of ADO in the assay (Supplementary Table 2), indicating that ADO is a competitive inhibitor for ATP, which corroborates with data from previous kinetic studies^19^. Overall the combination of the structural and kinetic data of the present study help to clarify some discrepancies in the literature about the kinetic mechanism and the structural models available for ADK of different species in its apo and complexed forms^2^. Although our present data do not completely discard the possibility of the existence of an additional regulatory adenosine-binding site in a different region of the enzyme^12–14,17^, it seems that, at least for the ADK from mammalian species, this is not the case.

In the mADK:ADO:ADP complex, the electronic density for ADO occupies the BS2 site formed by residues Asn30, Leu32, Leu56, Asp34, Gly79, Gly80, Ser81, Asn84, Cys139, Ala152, Leu150, Leu154, Phe186, Gly313, Asn312 and Asp316 (Supplementary Fig. 3b). In this complex, ADO shows a glycosidic torsion angle of −123.3° and a O4′-endo sugar pucker. The adenine ring displays a stacking interaction with Phe186, a hydrogen bond with Ser81 (N3-N), and interactions through water molecules with Phe186 (O) and Asn30 (OD1). Several hydrogen bonds anchor the ribose moiety: the O2′ and O3′ atoms bind to the side chain of residues Asp34, Gly80, and Asn84, while the O5′ atom interacts with the side chain of the catalytic residue Asp316. In this complex, ADP occupies BS1. This site arises from the edges of β strands β11, β12, β13, β14 and α helices α12 and α13 on the enzyme surface. The adenine group of ADP binds to Gln305 through a hydrogen bond and to Asp302 through a water-mediated interaction. Val300, Ile308, Ile347, Ala343 and Ala314 complete a hydrophobic cluster around the adenine ring (Supplementary Fig. 3c). The sugar ring does not interact directly with the enzyme, but it establishes hydrogen bonds with the solvent molecules through its O2′ and O3′ atoms. The phosphate tail of ADP interacts with the enzyme through an extensive network of direct and water-mediated hydrogen bonds. The O2A atom of the α-phosphate interacts with Thr281 via O^γ1^, Gly283 via N and Mg^2+^. Water molecules and Mg^2+^ mediate additional contacts with the α-phosphate and β-phosphate groups. The β-phosphate also interacts directly with mADK through hydrogen bonds with Arg148 via NH1-O1B from the lid domain, with Asn239 via ND2-O1B, and Gly315 via N-O3B from the large domain. Additional interactions between β-phosphate, Mg^2+^ and water molecules further confer stability to ADP binding to mADK.

### Conformational differences between the binary and the ternary mADK complexes

**Figure 3:**
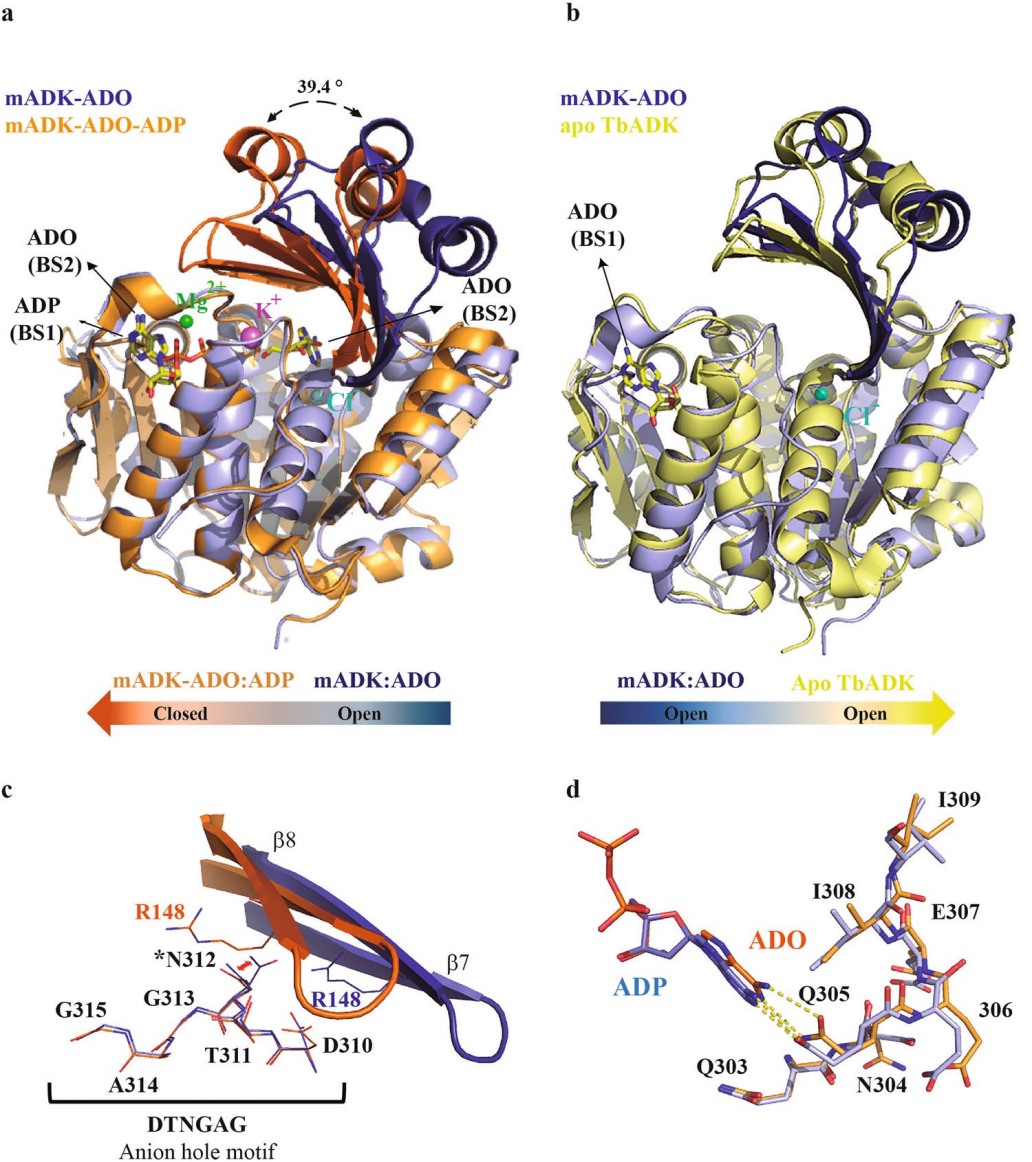
The mADK lid domain dynamics. (**a**) Superposed crystal structures of mADK binary (blue) and ternary complex (orange). The lid domain moves 39.4° over the large domain, causing the transition between a fully open and a closed conformation. The Mg^2^^+^ (green spheres) and K^+^ (purple sphere) ions were observed only in the ternary closed complex. The substrates are shown as sticks with carbon, oxygen, nitrogen and phosphorus atoms colored as yellow, red, blue and orange, respectively. (**b**) Superposed mADK binary complex (blue) and apo *T. brucei* apo form structure (PDB code: 4N08) (yellow). The lid domains of both structures are in the fully open conformation. (**c**) Superposed lid domain of the binary (blue) and ternary mADK (orange) complexes. The rotation of the lid domain results in the translocation of Arg148 residue about the active site, being able to interact with the β-phosphate of the ADP (ADP not shown). The anion hole residues (DTNGAG) are shown in sticks in both complexes. The asterisk highlights the Asn314 that exhibit a different conformation at an open lid form (binary complex) in relation to closed lid domain (ternary complex). (**d**) Influence of the potassium ion on the ATP P-loop residues conformation (Glu303-Ile309), continuous segment to anion hole. The Glu305 residue shows a distinct conformation in the ternary complex leading to a different position of the ADP adenine ring.

Crystal structures of ADK from different species have been determined and demonstrate a large extent of structural conservation, despite the considerable sequence diversity^16,17,20–24^. From these data arises a model in that the binding of ADO to the BS2 site induces a large-scale bending motion of the lid domain toward the large domain, resulting in a “closed” conformation in the active state. Subsequent formation of an anion hole, induced by the binding of ATP, completes the structural requirements for catalysis^23,25^. The superposition of the binary and ternary mADK structures highlights the large-scale conformational changes expected for the transition of a fully open to a closed form of the enzyme (Fig. 3a). The least-square fitting over all α-carbon atoms resulted in an r.m.s.d deviation between the structures of 3.32 Å. The ternary complex is in a closed conformation with the two domains moving by up to 39.4^°^ compared to the binary complex, which assumes a conformation that is reminiscent of the apo form of ADK from T. brucei^18^ (PDB: 4N08) (Fig. 3b). Besides, we found localized differences between the binary and the ternary complex, which show how binding to the substrates and the changes in closed conformation prepare the enzyme for catalysis (Fig. 3c). In the binary complex, Asn312 (which is an integral part of the anion hole) appears in two alternate conformers as a function of the torsion angle Ψ1 (gauche^+^ and trans conformations). However, in the closed ternary complex, it only adopts the trans conformation, which results from steric impositions by the C_β_, C_γ_, and C_α_ of Arg148 of the lid subdomain. This residue is brought to interact with the β-phosphate of ADP in the closed conformation, which perturbs the segment comprising the Gln303-Ile309 sequence adjacent to the anion hole segment (Fig. 3d). Interestingly, these changes appear to coincide with the presence of K^+^ between the α_11-12_ and the C-terminal loops.^.^

### Atomic detail of the potassium-binding site in mADK

**Figure 4:**
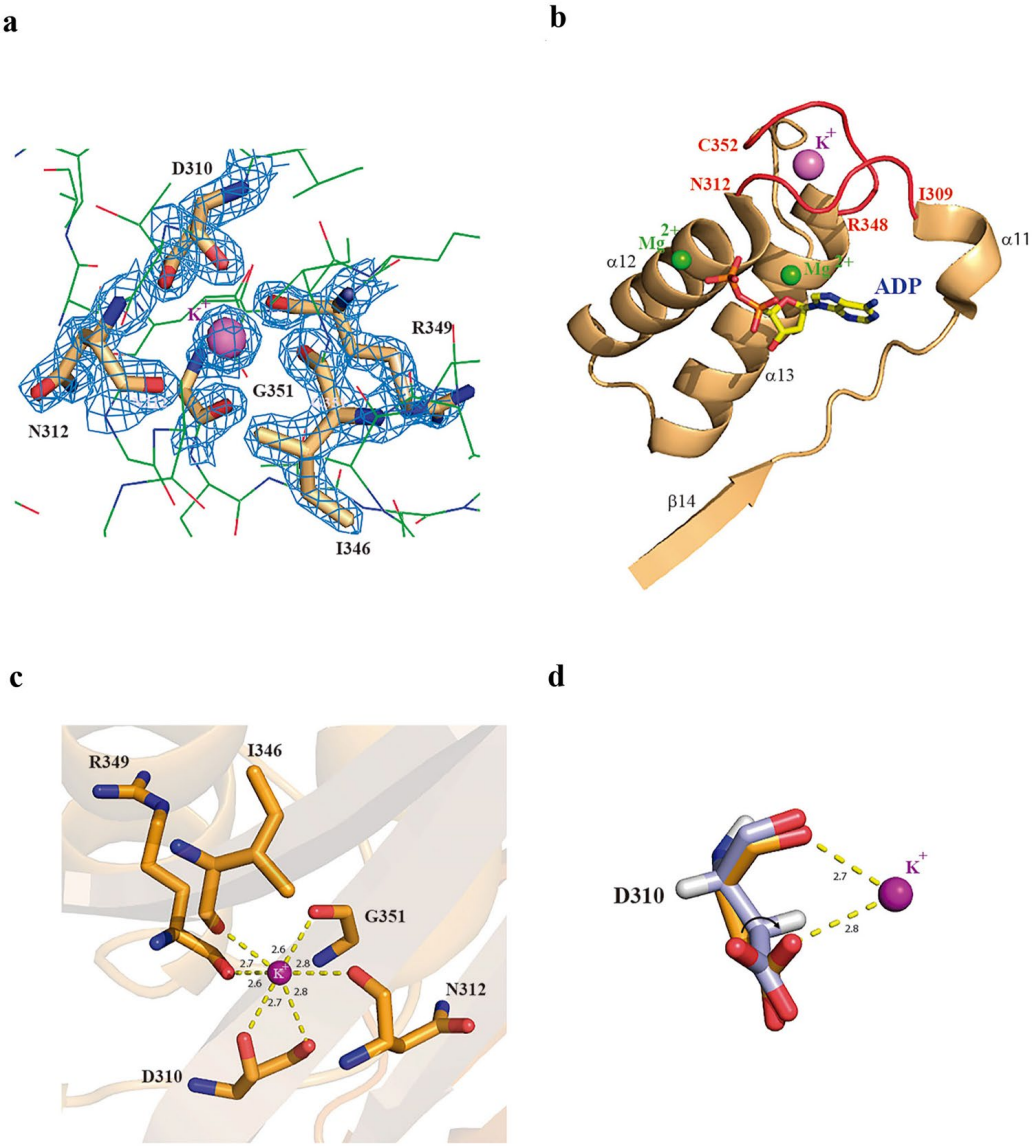
Putative K^+^ binding site in the mADK. (**a**) F_0_-F_c_ electron density of the K^+^ binding site. **(b)** The loops (colored in red) are surrounding the K^+^ binding site. Loops Ile309-Gly312 (α11 and α12 loop) and Arg348-Cys352 (C-terminus), adjacent to the BS1 pocket. The ADP molecule is shown as sticks with carbon, oxygen, nitrogen and phosphorus atoms colored in yellow, red, blue and orange, respectively. Two Mg^2+^ (green spheres) and K^+^ (purple sphere) ions are shown in the figure. **(c)** K^+^ is hexacoordinated by the backbone carbonyl oxygens of residues Asp310, Asn312, Ile346, Arg349 and Gly351. **(d)** Asp310, residue belonging to the anion hole, presents a distinct rotameric conformation in the K^+^ -bound form (mADK ternary complex) and an additional bond is observed (OD1-K). In the absence of K^+^ (mADK binary complex) this residue is rotated.

In this study, the high-resolution data along with coordination geometry analysis of the ternary mADK crystal structure enabled the identification of an atomic density corresponding to a K^+^ in the ADK structure (Fig. 4a). The K^+^ binding site locates in the large subdomain surrounded by two loops that span from residues Ile309 to Asn312 (α11 and α12 loop) and residues Arg348 to Cys352 (C-terminus), adjacent to the BS1 pocket (Fig. 4b). The ion adopts an octahedral geometry with the backbone carbonyl oxygen of residues Asp310, Asn312, Ile346, Arg349 and Gly351 (Fig. 4c). Besides, the critical residue for K^+^ coordination, Asp310, contacts the ion via main and side chains and presents a distinct rotameric conformation in the K^+^ -bound form. In the absence of K^+^ (mADK binary complex) this residue is rotated away from the ion (Fig. 4d). Of note, this is the last residue in the conserved anion hole sequence, which is postulated to deprotonate the alcohol function during catalysis. Similar monovalent cation binding sites have been reported for members of the ribokinase family^26^, suggesting a conserved influence of cation interaction structure on the formation of the anion hole in ADK.

### Potassium modulates mADK activity

**Figure 5:**
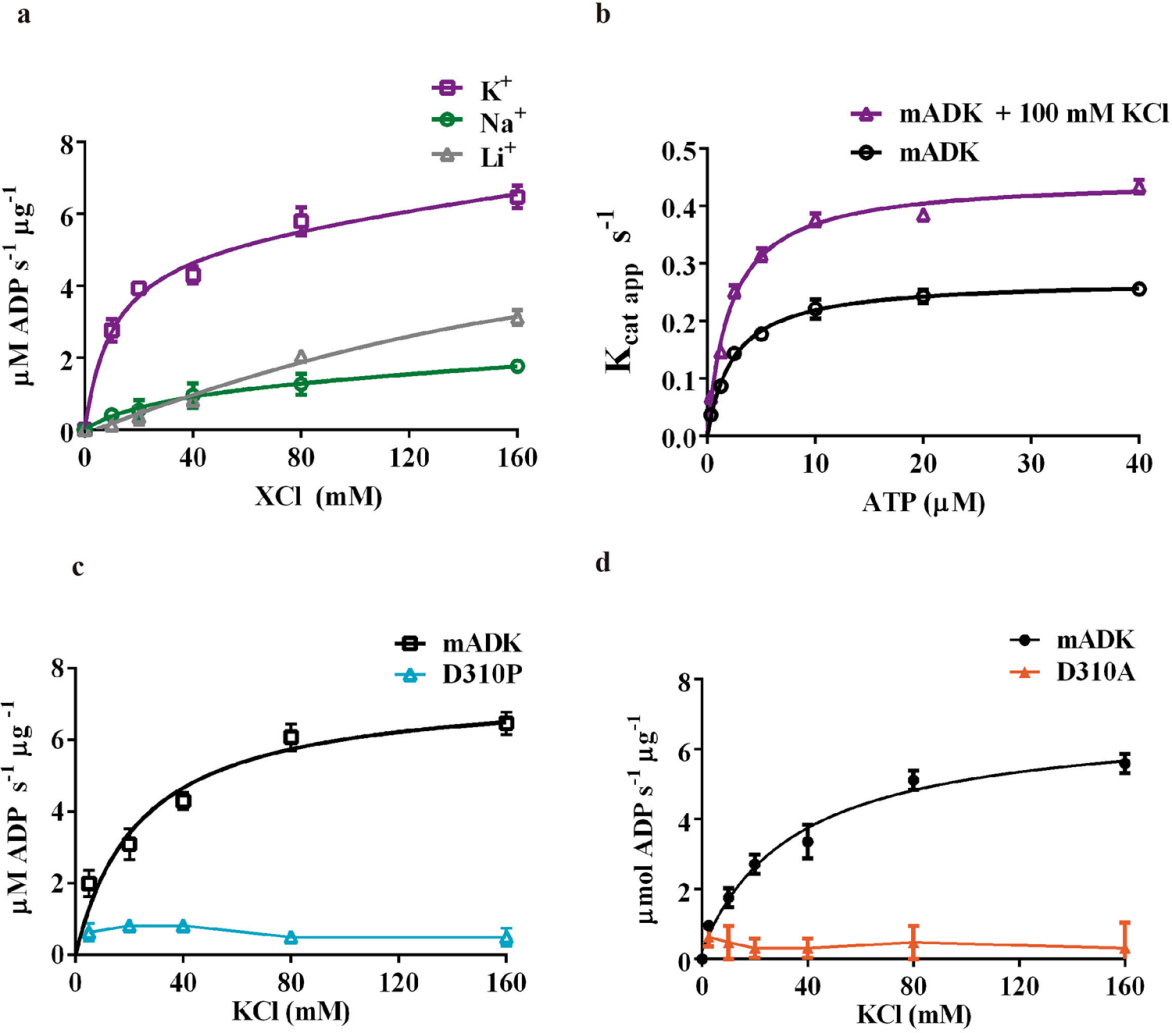
Activation of mADK by K^+^. (**a**) mADK kinase activity in the presence of chloride salts (KCl, NaCl and LiCl). The initial rate of the reaction of mADK was measured varying chloride salts from 0 to 160 mM. The reaction conditions were, 15 nM mADK, 20 µM ATP and 10 µM ADO. *K*_d_ average value obtained was 10.4 ± 2.30 mM and was estimated by one site total equation fitting by Prism 6.1 software (R square of 0.988 (K^+^)). There was no coherent fit for sodium and lithium salts. **(b)** *K*cat curves performance in the presence and absence of potassium. Initial rate of mADK reaction was measured in function of ATP in the presence (purple line) or absence (black line) of 100 mM potassium. Apparent *K*cat values were obtained by the *V*max (µM ADP. s^-1^)/ [mADK](µM). Continuous line represents the Michaelis Menten model fitting. Kinetic constants are shown in the Supplementary Table 3. **(c)** Effect of the D310A and **(d)** D310P mutations in the activation of mADK by K^+^. Both mutants abolished the mADK activation by the K^+^ ion.

The role of K^+^ in the mADK catalytic activity was determined by measuring the initial rate of the reaction as a function of chloride salt concentration in the assays (i.e., KCl, LiCl, NaCl) (Fig. 5a). Basal enzyme activity of mADK was still detected under conditions of zero K^+^ concentration in the buffer assay, suggesting that the K^+^ ion is not needed for the basal activity of this enzyme, similarly to what was previously suggested by studies performed in extracts of human placenta^27^. However, given the difficulties to completely eliminate the K^+^ ion in the biological samples (either from E. coli or other sources), one cannot be sure that K^+^ is not necessary for the basal activity of ADK. In the samples of purified E. coli recombinant mADK, the K^+^ ion increased the initial rate of the reaction of mADK by 2.5-fold with respect to a buffer without K^+^. The dependence of mADK activation on K^+^ is saturable, with maximal kinase activity observed at 40 mM KCl. These data are well described by an equation derived from a one-site binding model. The estimated *K*_d_ of 10.4 mM suggests that at the physiological concentration (140 to 150 mEq/L), K^+^ fully activates the enzyme. The effects of Na^+^ and Li^+^ were also measured to ensure that the effects of the monovalent cations on the mADK activity were limited to K^+^ and were not dependent on an ionic strength effect. We observed no significant change in the mADK activity with increasing concentrations of NaCl or LiCl. To address the mechanism behind the activation by K^+^, we characterized the enzymatic activity of mADK as a function of the ATP concentration in the presence or absence of 100 mM KCl (Supplementary Table 3). There was a two-fold increase in the apparent *K*_cat_ values (*K*_cat_^0^ = 0.25 s^−1^ versus *K*_cat_^*K*+^ = 0.46 s^−1^), while the apparent *K*_m_ remained almost the same with and without *K*^+^ (*K*_m_^0^ = 2.4 µM versus *K*_m_^*K*+^ = 2.1 µM). In fact, the addition of K^+^ to the reaction duplicated the catalytic efficiency (*K*_cat_/*K*_m_) from 0.1 to 0.22 s^−1^/µmol (Fig. 5b). Next, we examined if the structural integrity of the K^+^ binding site would be critical for the influence of this ion on mADK activity. Asp310 was targeted for mutation because structural analysis indicated that this residue is a crucial constituent of the loops that coordinate K^+^ binding to mADK (Fig. 4c). We performed mutations D310A, which substitute Asp310 by a neutral residue, and D310P because the Pro residue would be expected to break two interactions and yet affect the volume of the K^+^ ion coordination sphere cavity. Of note, as shown in the Supplementary Fig. 4a to d, all biophysical data supported that the D310A and D310P are well folded and still proficient at the catalytic point of view (Supplementary Table 1), but with reduced affinity (Supplementary Fig. 4e to h) to ATP (Km). As expected, both mutations completely abolished the sensitivity of the enzyme to the K^+^ (Fig. 5c,d), which is likely associated to the inability of the enzyme to be activated through conformational transitions induced by the binding to the K^+^ ion, similarly to other members of ribokinase family^26,28^.

### Wild-type mADK and D310P mutant ATP interaction by NMR investigation

To further investigate the impact of the D310P mutation on the enzyme interaction with ATP, we next used two- and three-dimensional NMR spectroscopy to compare the wild-type and the D310P mutant of mADK. 3D NMR experiments used U-labeled mADK for backbone sequential resonance assignments. From the expected 331 peaks, we observed 209 peaks in the ^15^N-^1^H HSQC, from which we attributed 200 peaks to ADK residues (Supplementary Fig. 5a). As shown in the Supplementary Fig. 5b, the missing signals are located at multiple stretches of residues. Residues represented in gray were those that were not found in NMR assays. During the titration steps in the ^15^N-^1^H HSQC protocol saturation of mADK by the ligand some signals appear in the spectra, possibly due to conformational changes, but the overlapped signals turn the spectra crowded and more difficult to attribute them. Moreover, the protein becomes unstable.

**Figure 6:**
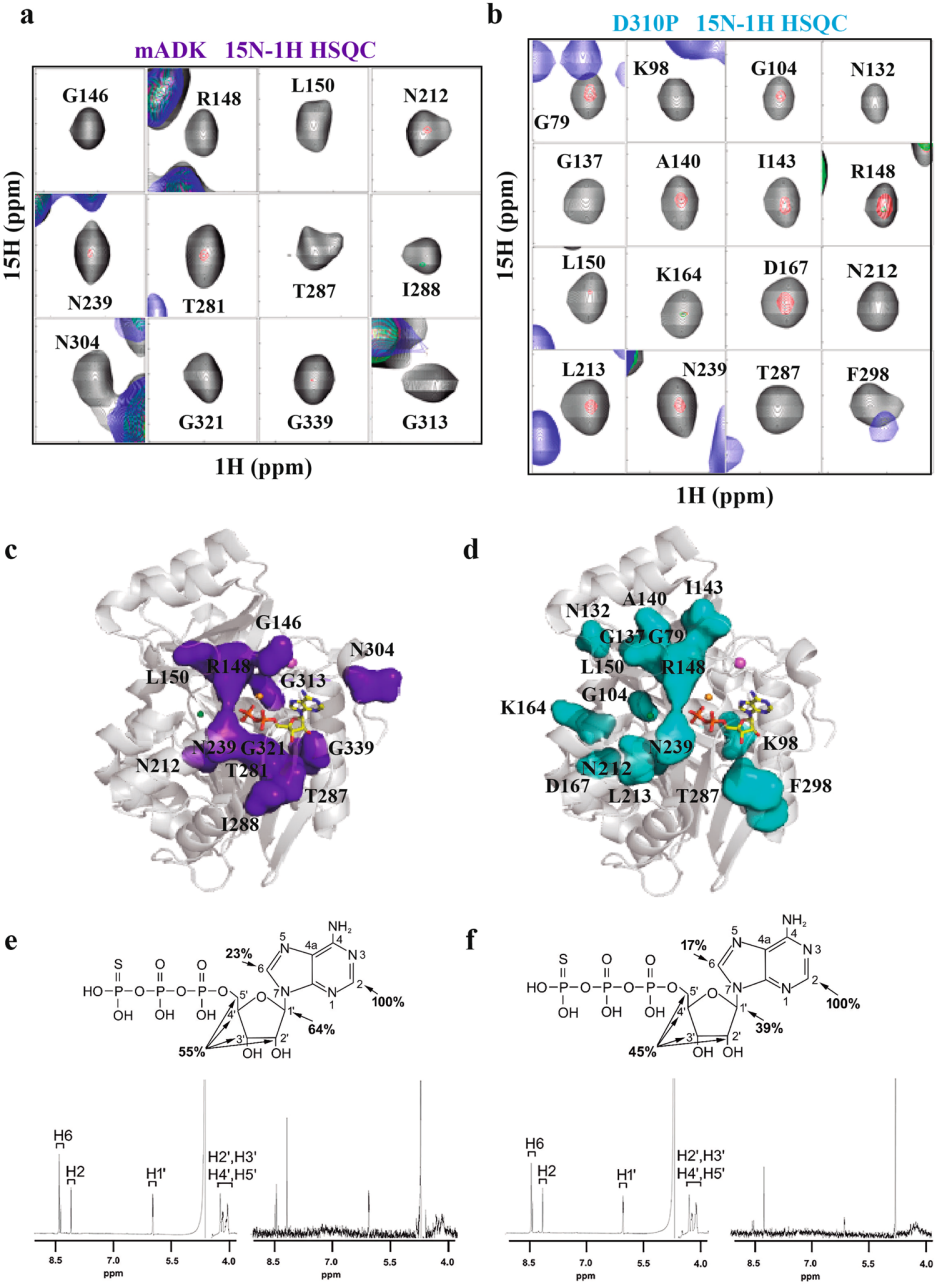
D310P mutation disrupts K^+^ sensitivity of mADK. (**a**) mADK and **(b)** D310P mutant analyzed by 2D ^15^N-^1^H HSQC. In the 2D ^15^N-^1^H HSQC spectra, the black, red, green and blue peaks represent chemical shifts changed ATPγS titration ratio of: free WT in black; 1:0.05 in red; 1:0.1 in green and 1:0.2 in blue. For the free D310P mutant, peak is black, followed by 1:0.1 in red, 1:0.2 in green and 1:0.5 in blue. **(c)** and **(d)** represent mapping of the broadened residues for mADK and D310P mutant respectively. Broadened residues are featured using as a model, the ternary mADK complex structure (PDB 5KB5). 1D ^1^H STD NMR spectra of ATPγS interaction with mADK **(e)** and D310P **(f**). Enhancements are referred to as 100% to the H-2 resonance of ATPγS. Upper part shows the ATPγS structure with the number for each proton and the % STD enhancement.

Individual 2D ^15^N-^1^H HSQC spectra from samples of ^15^N-labeled wild-type and D310P mutant mADK were recorded in titration series with adenosine triphosphate-gamma-S (ATPγS) for 8 hours, using a 600-MHz spectrometer with a cryoprobe. The proteins were stable under the temperature and buffer conditions of the experiment, as revealed by the analysis of the spectra from the reference samples. We focused on residues around the BS1 site, which showed similar spectra in the reference samples of the wild-type and D310P mutant mADK. Comparing the signals from both wild-type (Fig. 6a) and the mutant D310P mADK (Fig. 6b), we found distinct changes in the 2D ^15^N-^1^H HSQC spectra during the initial steps of the interaction with ATPγS. Analysis of the spectra obtained from the titration experiments of the wild-type mADK indicated exchange broadening of residues Gly146, Arg148, Leu150, Asn212, Asn239, Thr281, Thr287, Ile288, Asn304, Gly313, Gly321, and Gly339 (Fig. 6c). However, the spectra of the D310P mutant showed changes restricted to the residues Arg148, Leu150, Asn212, Asn239, and Thr287. Besides, signals from many other residues such as Gly79, Lys98, Gly104, Asn132, Gly137, A140, Ile143, Lys164, Asp167, Leu213, and Phe298 underwent a reduction in intensity (Fig. 6d). The results showed that ATPγS caused significant chemical shift changes in the wild-type mADK, especially in the residues around the BS1 site. However, the D310P mutant did not present substantial exchange broadening in the residues adjacent to the BS1 site (Gly146, Asn304, Gly321, Gly313 and Gly339). It appears, therefore, that the integrity of the K^+^ binding site is crucial to stabilizing the BS1 site, favoring the nucleotide interaction. Considering that D310P mutation might cause ATP binding to BS2, we show in Supplementary Fig. 6a a rotated pose to highlight the residues of the mutant mADK that are affected during ATPγS titration. As noted, residues of the BS2 (shown in orange) (Supplementary Fig. 6b) remained unchanged during ATP titration, indicating that the D310P mutation does not predispose the interaction of ATP to the BS2.

Also, 1D ^1^H STD NMR spectra were obtained for the wild-type mADK (Fig. 6e) and for the D310P mutant (Fig. 6f) with ATPγS (1:400) to assess the modifications in the substrate that result from the interaction with mADK. Signals from ATPγS H-2 were the ones that underwent the most substantial increase in intensity in the presence of either the wild-type or the mutant mADK. However, the enhancement of the signal (%STD) for the other protons was higher for the wild-type than those for the mutant mADK. The signal-to-noise ratio to the acquisition of the ^1^H STD NMR experiments demonstrated a value of approximately 40% lower for the mutant. In the case of ^15^N-^1^H HSQC titration, a greater amount of substrate was needed to see an initial backbone amide group cross-peak modification. Thus, 1D ^1^H STD NMR indicate that the D310P mutant has an unfavorable interaction with ATPγS relative to the wild-type mADK, which agrees with the kinetic data showing an increased Km for ATP in the D310P mADK.

### Depletion of cellular K^+^ is accompanied by ADK inhibition

**Figure 7:**
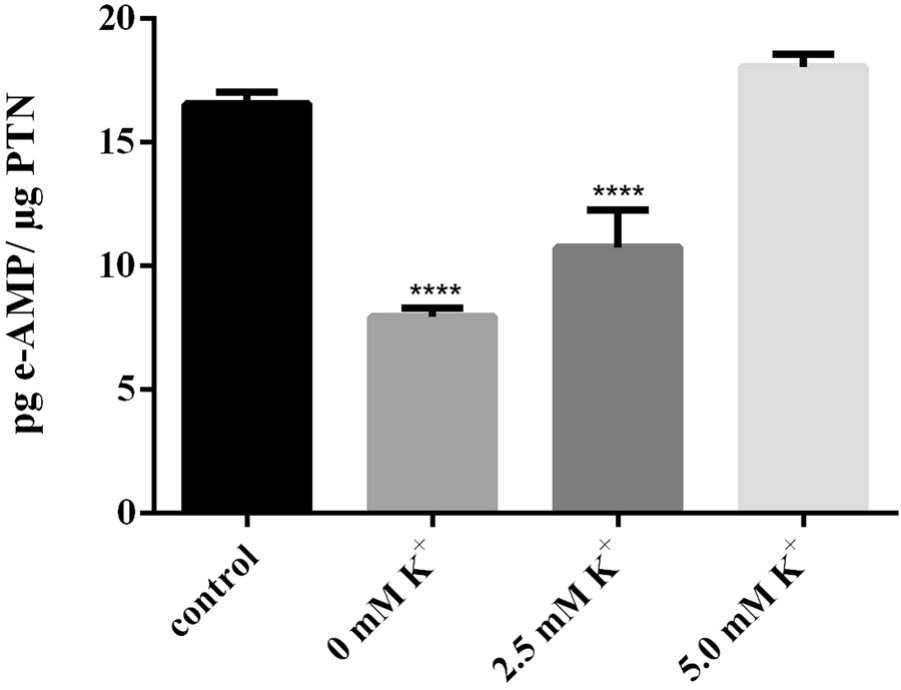
K^+^ depletion reduces the mADK activity in cells. HEK293 monolayers were subjected to hypotonic shock by 30 minutes followed by incubation in an isotonic K^+^ -free Buffer. After this, the cells received K^+^ -Buffer (0, 2.5 and 5 mM KCl) and the control cells received a DMEM medium. The cells were remained by1 hour under each evaluated condition. After the treatments, the cells were lysed and the kinase activity (pg e-AMP/ µg PTN) measured by HPLC quantification analysis. Statistical analysis: one-way ANOVA. Post hoc test: Bonferroni (*For control P < 0.05). (Error bars represent 6 SD of the mean).

To test whether fluctuations in the intracellular levels of K^+^ could influence the activity of ADK *in vivo*, we performed experiments in HEK297 cells subjected to a previously reported experimental protocol of depletion^29^, followed by restoration of intracellular K^+^. In agreement with the *in vitro* data, ADK activity was reversibly inhibited when the K^+^ concentration in the culture medium was reduced, while the activity was restored when the K^+^ levels were gradually increased to normal levels (Fig. 7).

## Discussion

ADK is regarded as a regulator of intra- and extracellular levels of ADO and adenylate pools^2^. The structural, kinetic and mutational results reported in this study reveal that K^+^ selectively activates mADK through an allosteric regulatory mechanism shared among the members of the ribokinase family. Also, we report that in cells, depletion of K^+^ inhibits ADK, suggesting a link between the intracellular levels of K^+^ and the regulation of ADO and adenylate pools through ADK.

Numerous results have demonstrated that many members of the ribokinase family of phosphotransferases can be activated by K^+^ ^25,26,28^. Structures of the *E. coli* ribokinase, *Salmonella enterica* aminoimidazole riboside kinase and *E. coli* phosphofructokinase-2 show a K^+^ or the equivalent Cs^+^ bound to a conserved site among the family members^28,30,31^. Characteristically, the K^+^ binding site of the ribokinase family locates between two loops of the large domain, immediately adjacent to an anion hole and the ATP-binding site^28^. From these studies, a model has been proposed in which the interaction of K^+^ with the residues of the loops results in conformational changes that assist the formation of the anion hole, which contributes to the catalytic transfer of the phosphate group from ATP to the substrate. This model can explain the enhanced activity of ribokinase family members upon binding of K^+^. Notably, although structures of ADK from different sources have been shown to possess the conserved structures of the K^+^ binding site and the anion hole sequence DTNGAG in the large domain, a definitive structural identification and biochemical characterization of ADK activation by K^+^ are shown here for the first time. The high-quality electron density maps of the mADK ternary complex enabled us to model a K^+^ between the structurally conserved loops, connecting the C-terminal extremity and α13 (loop 1) to other loop formed by α11 and α12 helices (loop 2). As in other members of the ribokinase family, this site is located nearby the ATP-binding site and the anion hole sequence. The model predicts a K^+^ ion hexacoordinated across the carbonyls of residues Arg349, Ile346, Asn312, Gly351 and both side and main chains of Asp310, with oxygen-K^+^ distances ranging from 2.6 to 2.8 Å (mean value of 2.7 Å). Asp310 is also an integral part of the anion role. It is interesting to note that the mADK binary complex, which lacks a bound ion in it, retains the structural features of the K^+^ binding site. However, superposition of the ternary and binary complexes shows that the presence of K^+^ perturbs the entire P-loop segment, comprising the Asn304-Ile309 sequence. These changes are presumed to influence the binding of ATP to its cognate site in the enzyme.

Our biochemical investigations demonstrate that K^+^ activates mADK with exquisite specificity over other metal ions such as Na^+^ and Li^+^. Consistent with the structural data, single mutations of Asp310 namely D310A and D310P, which disrupt interactions with K^+^, abolished the activating effect of K^+^ on mADK. Notably, the dependence of mADK on K^+^ is saturable with maximal kinase activity observed at a K^+^ concentration of 40 mM, while the activity is very low in the absence of K^+^. The estimated *K*_d_ of 10.4 mM suggests that, under the physiological conditions, K^+^ fully activates the enzyme. Also, the results point to an influence of K^+^ on the enzyme turnover, as indicated by the two-fold increase in the values of apparent *K*_cat_, while the affinity for ATP, as assessed by the *K*_m_, was unaffected by the presence of excess K^+^ in the reaction. Thus, the mechanism by which K^+^ activates ADK is likely related to the enhanced efficiency of the phosphate transfer, instead of the affinity of ADK for ATP, as has been suggested for other members of the ribokinase family^28,30^.

Discrepancies regarding the effect of K^+^ ion on the kinetics of mADK showed here with data reported for other ion-sensitive enzymes may lead to uncertainty about the functional importance of the K^+^ ion to the mADK function. For instance, a 60-fold increase in the enzyme activity induced by increases in the K^+^ ion was previously reported to the *E coli* ribokinase^28^. Similarly, a 10-fold increase in the K^+^ ion was reported to enhance the *M tuberculosis* ADK activity by 3-fold^32^. The reasons for the apparent discrepancies between our data and those reported in the literature are unclear, however they can likely be due to differences in the methodologies, study designs and source of samples (e.g. purified enzyme vs cell extracts) utilized in the various studies. For instance, many of the studies in the literature lack complete kinetic data, which make the comparisons between the studies a difficult undertaking.

It is also important to note that the K^+^ ion is not apparently required for basal mADK, as some level of the enzyme activity is still present in the assays performed without addition of K^+^ to their buffer composition. Similarly, previous data obtained in samples of human placenta^33^ indicate that the K^+^ ion seems not be required for basal activity of human ADK. These data may contradict the notion of a major regulatory role for the K^+^ ion on ADK. However, one cannot be sure that the K^+^ ion is completely eliminate from the biological samples, even after prolonged dialysis against zero K^+^ buffers as discussed below.

Another interesting finding of our study is that cellular depletion of K^+^ reduces the activity of ADK. It is worth to note that reductions of intracellular K^+^ to very low levels, obtained by a hypotonic zero potassium solution, were sufficient to reduce the activity of intracellular ADK by approximately 80%. This protocol is expected to reduce the intracellular K^+^ by approximately 70% of the basal normal levels, which would bring the intracellular levels to 50 mEq/L^29^. Somehow this may contradict the biochemical data, as a *K*_d_ of 10 mM would preclude ADK from being inhibited by the diminished K^+^ concentration, meaning that the enzyme would be present only in the bound form. However, one must consider that there is considerable uncertainty as to how much of K^+^ exists in the free rather than the bound state, as the cytoplasm contains numerous species such as nucleic acids, phospholipids, proteins, and small-molecule metabolites with an affinity for K^+^. In fact, ^39^K NMR data suggest that up to 50% of cytoplasmic K^+^ may be in the bound state^34^. The finding that ADK is inhibited under K^+^ depletion conditions is particularly interesting, because ADK is inhibited during hypoxia, a situation that is accompanied by a rapid depletion of intracellular K^+^. Importantly, inhibition of ADK during hypoxia has been postulated to contribute significantly, albeit not exclusively, to the early accumulation of ADO during hypoxia^3,4^. In fact, a pathophysiological hallmark of the ischemia/hypoxia is the early increase in ADO signaling led by its accumulation in both the intracellular compartment and extracellular space^35^. Thus, our present data encourages us to speculate that inhibition of ADK by low levels of K^+^ serves to regulate the intracellular levels of ADO under pathophysiological situations like ischemia and hypoxia.

## Methods

### Engineering of mADK variants

Murine *ADK* was amplified from *Mus musculus* cDNA library using primers containing *B*am*H*I and *H*indIII restriction endonucleases sites. PCR product was cloned into a pET28a expression vector comprising a sumo-cleavable N-terminal His6-tag to produce two m*ADK* WT constructs (denoted Full_m*ADK* Met1 for His362 and Δ19m*ADK* Leu20 – Phe360, residues respectively). D310A and D310P site-directed mutagenesis were performed on pET28a-full_m*ADK* using the QuikChange II Xl (Stratagene) method, DNA was transformed into DH5α competent cells and the mutations were confirmed by DNA sequencing.

### Expression and purification

mADK and mutants were produced in *E. coli* BL21(DE3) strain (Novagen). The expression conditions were 0.5 mM isopropyl β–D- thiogalactopyranoside at 293 K for 18 hours. Frozen pellets were thawed, sonicated and clarified by centrifugation. The purification steps comprised the nickel-affinity with a HisTrap 5 ml (GE Healthcare) employed buffer A (50 mM sodium phosphate buffer, pH 7.8, and 300 mM sodium chloride), buffer B (50 mM sodium phosphate buffer pH 7.8, 300 mM sodium chloride, 10% *v/v* glycerol and 10 mM imidazole) and the elution step in buffer C (50 mM sodium phosphate buffer, pH 7.8, 300 mM sodium chloride, 10% *v/v* glycerol and 500 mM imidazole). After obtaining of fractions containing mADK_HisTAG, a cleavage of the His6-tag was performed during dialysis step into Buffer B, using Ubl-specific protease at 4 °C over 16 hours. Adsorption of the cleaved 6His-tag, was performed by an affinity purification step by Ni^2+^ resin and the mADK without 6His-tag was obtained in a flow- through fraction. The protein was concentrated using Amicon ultrafiltration cell (10 K MW cutoff membrane – EMD/Millipore). The protein was submitted to a size-exclusion chromatography step (Hiload 16/60 Superdex 75 prep grade) in buffer D (20 mM sodium phosphate buffer pH 7.8, 300 mM sodium chloride, 10% *v/v* glycerol, 5 mM imidazole and 1 mM dithiothreitol). mADK WT was concentrated to ~18 mg ml^−1^ for crystallization trials.

### Biophysical characterization of the mutants

Secondary structure prediction and thermal denaturation of the D310A and D310P mutants were performed by circular dichroism in Jasco J810 CD spectropolarimeter. The wild-type enzyme was used as default. Spectra of 5 µM of mADK WT and mutants were recorded in a buffer 20 mM Na_2_HPO_4_ pH 7.5 and 10 mM NaCl between 198 and 260 nm in a 1 mm optical path length cuvette. The denaturation was monitored at 222 nm with a temperature increase of 1 °C increments with 2 minutes equilibration time between data points. The percentage of unfolded protein was calculated by assuming samples were completely folded and unfolded between 30 °C and 90 °C, respectively.

### Crystallography

mADK WT was crystallized by the sitting drop vapor diffusion method in droplets composed of one part protein solution (18 mg/ml) to one part reservoir solution (lithium sulfate 0.2 M, sodium acetate 0.1 M and PEG 400 50%) for the mADK:ADO complex and one part reservoir solution (PEG 400 40%, PEG 1000 15% and sodium di-potassium phosphate) for mADK:ADO:ADP. Crystals appeared in approximately 7 days at 18 °C. The complexes were prepared by previous incubation with 2.5 mM adenosine (ADO) and adenosine diphosphate (ADP) at 4 °C for 16 hours.

### Data collection, processing and refinement

Data were collected at the MX2 beamline (LNLS, Campinas, Brazil) using 1.458 Å wavelength X-ray. As many as 360 images were obtained using an oscillation angle of 0.5° and an exposure time of 20 s for the complex mADK-ADO and 30 s for the other set of data. The detector distance was set to a maximum resolution of 1.2 and 1.8 Å for mADK:ADO and mADK:ADO:ADP, respectively. A charge-coupled device (CCD), MAR Mosaic 225 mM (MAR Research) was used to record the intensities. Data were indexed and scaled using Denzo and Scalepack from the HKL-2000 package^36^. The structure of mADK:ADO was solved by molecular-replacement calculations using the program Phaser^37,38^ with the HsADK as a template (PDB entry 1BX4)^16^. The mADK refined coordinates from the mADK:ADO crystal was further used to find an MR solution for the mADK:ADP complex. Both structures were refined using REFMAC5^39^. In the mADK:ADO structure, the thermal parameters of all non-hydrogen atoms were treated anisotropically, whereas, in the mADK:ADP, TLS parametrization was applied. Following each cycle of refinement, the model was manually adjusted to correspond to computed weighted (2Fo-Fc) and (Fo-Fc) electron density maps using the program Coot^40^. Water molecules were manually added at positive peaks above 1.8 Å in the difference Fourier maps, taking into consideration hydrogen-bond distances. The refined structures were evaluated using the program MolProbity^41^. Refinement statistics are summarized in Table 1. The images were generated using PyMol Molecular Graphics System, version 2.1.1 Schrödinger, LLC.

### Kinase activity assay

mADK activity was performed by monitoring the resorufin fluorescence produced by a coupled kinases system based on the ADP consumption^42^, catalytic product of the mADK reaction. The ADP enters as substrate into a coupled assay that employs Pyruvate kinase (20 UI/mL), Horseradish peroxidase (2 UI/mL), Pyruvate oxidase (1 UI/mL), phosphoenolpyruvate (0.3 mM) and cofactors thiamine pyrophosphate (100 µM), flavin adenine dinucleotide (10 µM), 10-acetyl-3,7- dihydroxyphenoxazine (35 µM) responsible to convert ADP in hydrogen peroxide, which in turn is oxidized to form resorufin. The fluorescence was quantified by 530–570 nm of excitation and emission at 590 nm in an *EnVision*® Multilabel Plate Reader (Perkin Elmer). The assays were performed at 303 K for 30–60 minutes. The kinetic parameters of the mADK WT were assayed employing 15 nM enzyme with serial dilutions to ADO or ATP (0 to 40 µM) fixing ATP (20 µM) or ADO (10 µM) in a reaction buffer 50 mM Tris-HCl pH = 7.5, 20 mM KH_2_PO_4_, 10 mM MgSO_4_ and 0.01% Triton X-100_._ Kinase activity for the D310A and D310P mutants, were performed with 150 nM enzyme, substrates varying ADO 0 to 80 µM (in the presence of 50 µM ATP) and varying ATP 0 to 320 µM (in the presence of 50 µM ADO) (Supplementary Table S1). All kinetic data were normalized extrapolating the values obtained by the standard ADP curve (RFU/s *versus* [ADP] µM). kinetic parameters were analyzed using the program GraphPad Prism 6.07 version with non-linear fitting substrate inhibition and the Michaelis-Menten equations.

### Potassium activation assay

Before kinetic activity measurements, mADK and the mutants (D310A and D310P) were exhaustively dialyzed against 30 mM Tris-HCl (pH 7.5). For the investigation of activation by monovalent cation, the following conditions were employed: 15 nM mADK WT with 10 µM ADO and 20 µM ATP, and for the mutants, was used 150 nM enzymes in the presence of 50 µM ADO and 50 µM ATP. The assay was performed in a reaction buffer without potassium, 50 mM Tris-HCl pH 7.5, 10 mM MgSO_4_ and 0.01% Triton X-100 using dose-response curve of KCl, LiCl or NaCl from 0 to 160 mM. The activity assays were measured at 303 K and the kinetic parameters were analyzed using the program GraphPad Prism 6.07 version with one site total curve fitting.

### NMR analysis

NMR data were collected at 298 K using an Agilent DD2 500 MHz or Inova 600 MHz spectrometer equipped with a triple-resonance (^15^N/^13^C/^1^H) cryogenic probe. NMR samples were prepared in a 20 mM sodium phosphate buffer, pH7.5, 50 mM sodium chloride, 40 mM potassium chloride, 2 mM magnesium chloride, 1 mM DTT containing 5% D_2_O/95% H_2_O. Spectra for sequential resonance assignment of mADK were acquired from 330 µL samples of 0.5 mM ^2^H/^13^C/^15^N protein using a Shigemi tube. Trosy version of the following experiments was recorded: HNCACB, HN(COCA)CB, HN(CO)CA, HNCA, HNCO and HN(CA)CO using standard methods for the assignment^43^. The specific binding of small molecule substrates to mADK or the D310P mutant was monitored by changes of signals of 220 μM ^15^N-labeled enzyme in 2D ^15^N-^1^H HSQC spectra by titration with the substrate. Also, the binding of small molecules was followed by ligand-detected saturation transfer difference (STD) experiments^44^. STD spectra were acquired using 600 µL samples of 1 μM unlabeled mADK or the D310P mutant in the presence of a 400-fold excess of ATPµS substrate with 2.5 s irradiation alternating between 0.5 ppm (on-resonance) and 30 ppm (off-resonance) using a train of 50 ms Gaussian pulses and 4096 transients. All spectra were processed using NMRPipe/NMRView^45,46^ and SpinWorks software.

### Intracellular potassium depletion

1 × 10^7^ HEK293 cells were grown in a monolayer, seeded into a petri dish (150 mm × 15 mm) containing 20 ml Dulbecco’s modified Eagle’s medium (DMEM) supplemented with 100 U/ml penicillin, 100 pg/ml streptomycin, 2 mM glutamine and 1 mM sodium pyruvate. Cells were grown at 310 K and 5%CO_2_. On day 3, cell growth reached 80% confluence, intracellular potassium was depleted by the Larkin method with modifications^29^. The medium was discarded off each plate and washed in 15 ml Buffer A (50 mM sodium-Hepes and 100 mM NaCl at pH 7.4). Each cell monolayer was subjected to 10 minutes incubation in 15 ml hypotonic medium (DMEM/ water, 1:1), followed by incubation during 30 minutes in an isotonic K^+^- free Buffer (50 mM sodium-Hepes, 140 mM NaCl, 1 mM CaCl_2_ and 0.5 mM MgCl_2_ at pH 7.4). The medium was removed and the monolayer incubated with the appropriate potassium concentration ((CT (DMEM), 0 mM KCl, 2.5 mM KCl and 5 mM KCl)) in 15 ml Buffer B (50 mM sodium-Hepes, 140 mM NaCl, 1 mM CaCl_2_ and 0.5 mM MgCl_2_ at pH 7.4) over 90 minutes. After incubation, the cells were collected and centrifuged (500 × g, 5 min, 298 K). An aliquot of the cells in each conditional was submitted to flow cytometry for counting and normalization. The lysate the cell lysates were subjected to ADK activity assays.

### Quantification and statistical analysis

GraphPad Prism 6.07 was used for graphics and analyses. Curve fitting for kinetic constants was performed using non-linear fit - equations choose one site total, enzyme kinetics to Michaelis-Menten or Substrate Inhibition.

### Accession Number

The atomic coordinates and structure factors have been deposited in the Protein Data Bank under accession codes 5KB5 and 5KB6.

## Electronic supplementary material

Supplementary information

Related Manuscript File

Related Manuscript File
