# Supplementary material for: Adenosine Kinase couples sensing of cellular potassium depletion to purine metabolism
Source: Sci Rep. 2018 Aug 10;8:11988. doi: 10.1038/s41598-018-30418-5 (PMC6086891; doi:10.1038/s41598-018-30418-5)
Supplement: Supplementary file 1 — Supplementary information [file 41598_2018_30418_MOESM1_ESM.docx]

**Supplementary information**

**Adenosine Kinase couples sensing of cellular potassium depletion to purine metabolism**

Renata Rocha de Oliveira^1^, Raphael Morales Neto^1^, Silvana Aparecida Rocco^1^, Maurício Luis Sforça^1^, Carla Cristina Polo^1^, Celisa Caldana Costa Tonoli^1^, Gustavo Fernando Mercaldi^1^, Artur Torres Cordeiro^1^, Mário Tyago Murakami^1^, and Kleber Gomes Franchini^1,2*^

1. Brazilian Biosciences National Laboratory, Brazilian Center for Research in Energy and Materials, Campinas, São Paulo, 13083-970, Brazil.
2. Department of Internal Medicine, School of Medicine, University of Campinas, Campinas, São Paulo, 13081-970, Brazil.

**corresponding author**:

Kleber G. Franchini, M.D. Ph.D.

Laboratório Nacional de Biociências, Centro Nacional de Pesquisa em Energia e Materiais, Rua Giuseppe Máximo Scolfaro 10000, CP 6192, 13083-970, Campinas/SP, Brazil, Tel 55-19-3512-1013, Fax 55-19-3512-1100.

Email: [kleber.franchini@lnbio.cnpem.br](mailto:kleber.franchini@lnbio.cnpem.br)

**Supplementary Figures and Tables legends**

# Supplementary Figure S1. (a) Structures of mADK in complex with adenosine and (b) mADK in complex with adenosine and ADP. The mADK is a monomer formed by two unequally sized domains (i.e., a large three-layer (αβα) sandwich subdomain and a small lid α/β subdomain). The α-helices and β-sheets were numbered sequentially from α1 to α13 and β1 to β14 according to HsADK^(^*^1^*^)^. ADO and ADP are shown as sticks with carbon, oxygen, nitrogen and phosphorus atoms colored in yellow, red, blue and orange, respectively. Spheres represent magnesium (green), chloride (pale cyan) and potassium (purple) ions. The mADK active sites are highlighted as BS1 (nucleotide site) and BS2 (nucleoside site), both located in a cavity of the large domain.

# Supplementary Figure S2. (a) Superposition of the mADK binary complex (PDB code:5KB6 shown in blue) and HsADK binary complex (PDB code: 1BX4 shown in grey) (RMSD overall 3.14Å, Lid domain 9.74Å and large domain 0.77Å). The figure shows the occupancy of BS1 of both mADK and HsADK by ADO. The mADK structure presented an additional α helix (α11) instead of a loop that appears in the HsADK. (b) Stereo view of the BS1 showing that ADO occupies a similar pocket in both mADK and HsADK structures. The residues were colored in gray and blue to HsADK and mADK, respectively.

**Supplementary Figure S3. (a)** Stereo view of the BS1 interaction with ADO identified at the mADK binary complex. A large hydrophobic cluster (light blue residues) surrounds ADO together with five coordinated water molecules (red spheres). **(b)** Stereo view of the BS2 interactions with ADO at the ternary complex. In this structure, the ADO molecule was identified in the active nucleoside site (BS2). **(c)** Stereo view of the BS1 interactions with ADP in the ternary complex. ADP directly contacts with two magnesium ions, previously known as cofactors of mADK and large hydrogen bonding network formed by eleven water molecules which anchor the phosphate tail to the active configuration for catalysis. The β-phosphate directly contacts Arg148 (highlighted in dark orange sticks) present in the lid domain.

**Supplementary Figure S4. (a)** Residual molar ellipticity measures of D310P mutant (cyan) and mADK wild-type (black) and **(b)** D310A mutant (orange) and mADK wild-type (black) evaluated by circular dichroism at 200 to 260nm. The protein samples were at a 5µM in a 10 mM phosphate buffer, pH 7 and 10 mM NaCl. **(c)** Thermal stability curves of the D310P **(d)** and D310A measured by CD. The molar ellipticity was assessed at 222 nm in temperatures varying from 30 to 80 °C. The melting temperatures corresponding to each mutant are indicated in figure. **(e)** The D310P and **(f)** D310A *K*m curve for ADO. The initial rate of the reaction was measured with 150nM enzyme (D310P or D310A), 50µM ATP and ADO concentration varying from 0 to 80µM. The kinetic parameters for ADO were obtained by substrate inhibition fitting model. **(g)** D310P and **(h)** D310A *K*m curve for ATP. The initial rate of the reaction was measured using 150nM of each mutant (D310P or D310A), 50µM ADO and ATP concentration varying from 0 to 320µM. For the ATP substrate, the mutants exhibited characteristic Michaelis Menten Kinetic. Kinetic constants for both ADO and ATP substrates are shown in the Supplementary Table 1.

**Supplementary Figure S5. (a)** TROSY 15N-1H HSQC spectrum of U-{15N, 13C, 2H}-labeled mADK recorded at 298K. Two regions of the spectrum are enlarged, and peaks are labeled with residue numbers. **(b)** Multiples stretches of the mADK residues sequence. Residues in gray were those that were not found in NMR assays by the 15N-1H HSQC protocol saturation of mADK by the γATP.

**Supplementary Figure S6. (a)** Stereo view of the mADK structure (gray; PDB:5KB5). Highlighted in cyan are shown the signals of residues that are broadened in the D310P mutant in comparison with the wild-type mADK after ATPγS titration by 2D ^15^N-^1^H HSQC. The rotated structure pose (90°) shows the location of the additional residues of D310P mutant that are also broadened after the ATPγS titration, which are shown to be located in regions that are distinct from the BS2. **(b)** The residues that compose the BS2 are shown in orange and they are not affected during the ATPγS titration.

**Supplementary Table 1.** mADK kinetic parameters.

**Supplementary Table 2.** mADK kinetic parameters for the substrate competition assay.

**Supplementary Table 3.** Kinetic parameters of mADK for ATP in the presence and absence of potassium.

**Supplementary Figure S1**


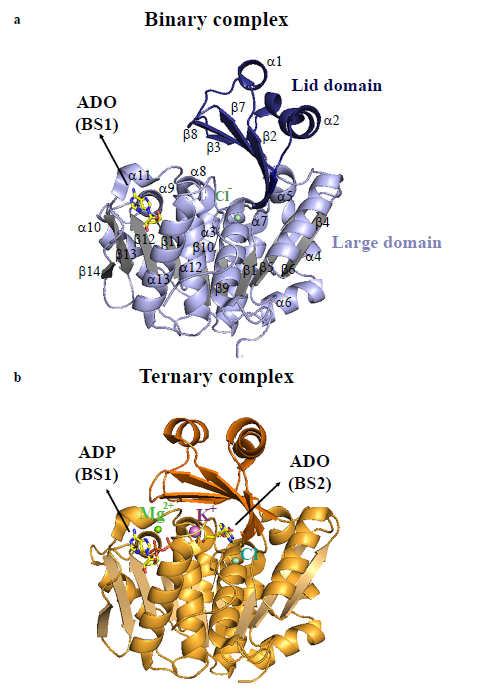


# Supplementary Figure S2

#
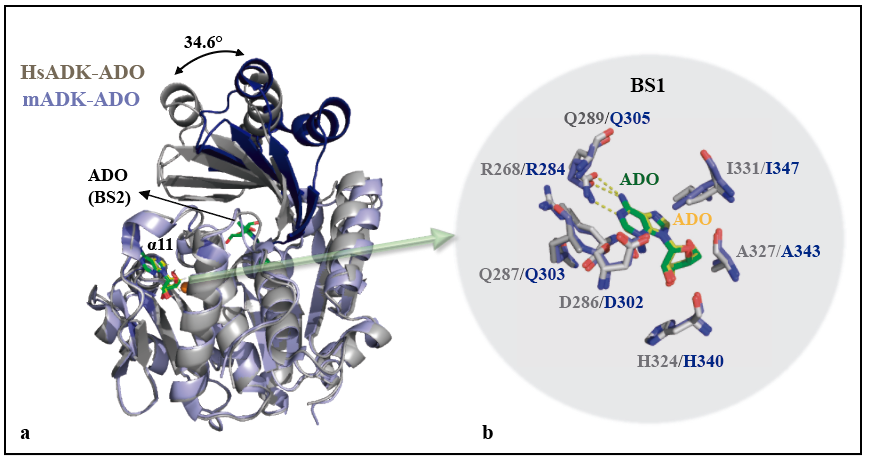


**Supplementary Figure S3**


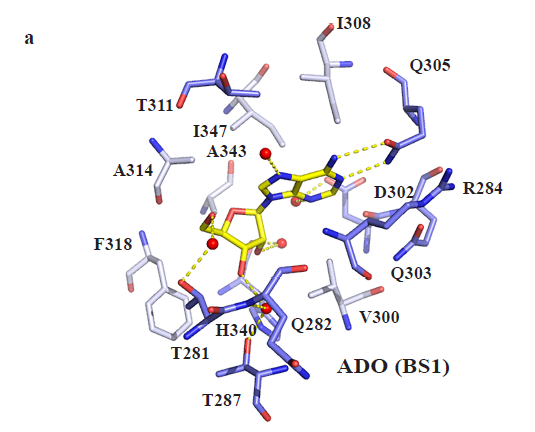


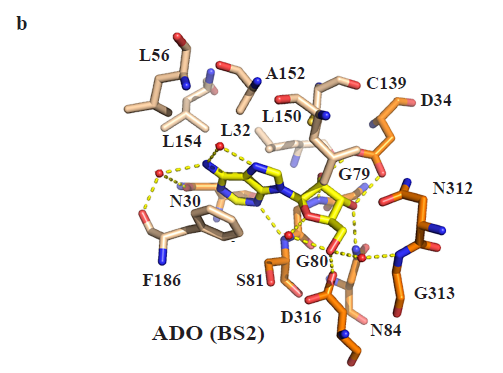


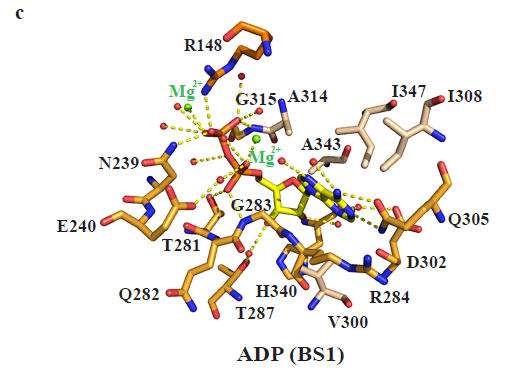


**Supplementary Figure S4**


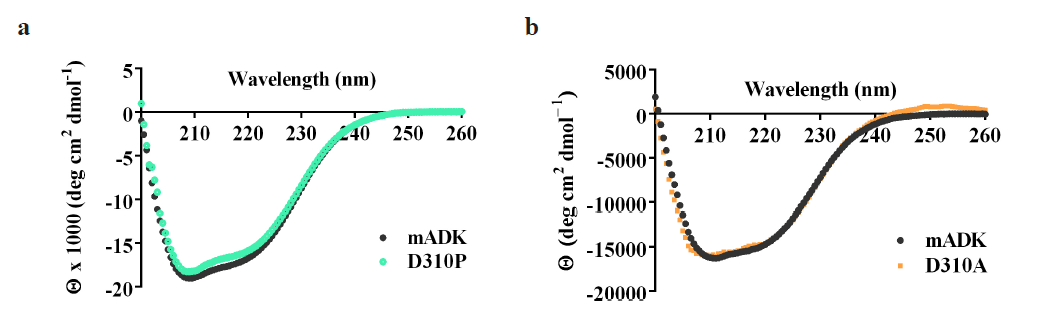


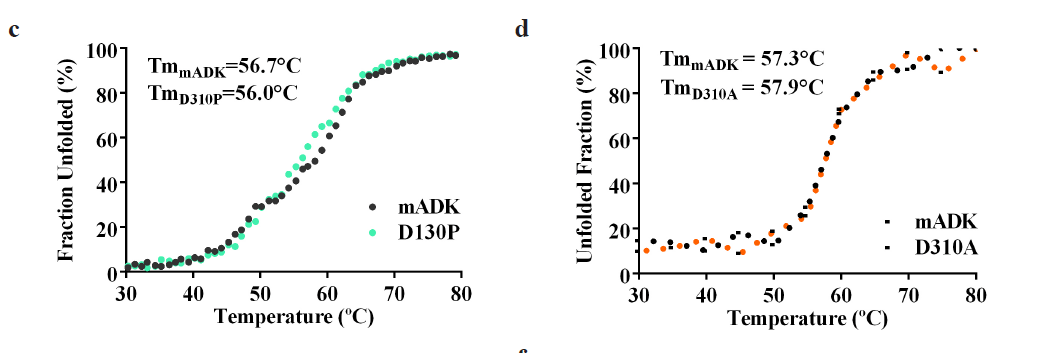


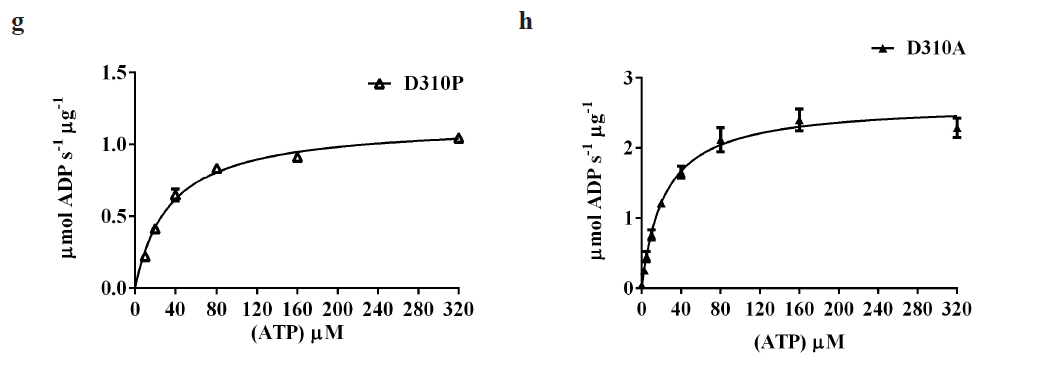

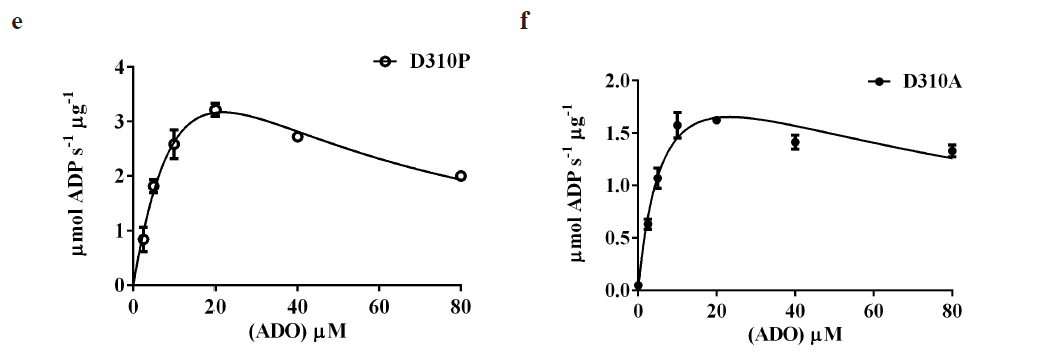


**Supplementary Figure 5**


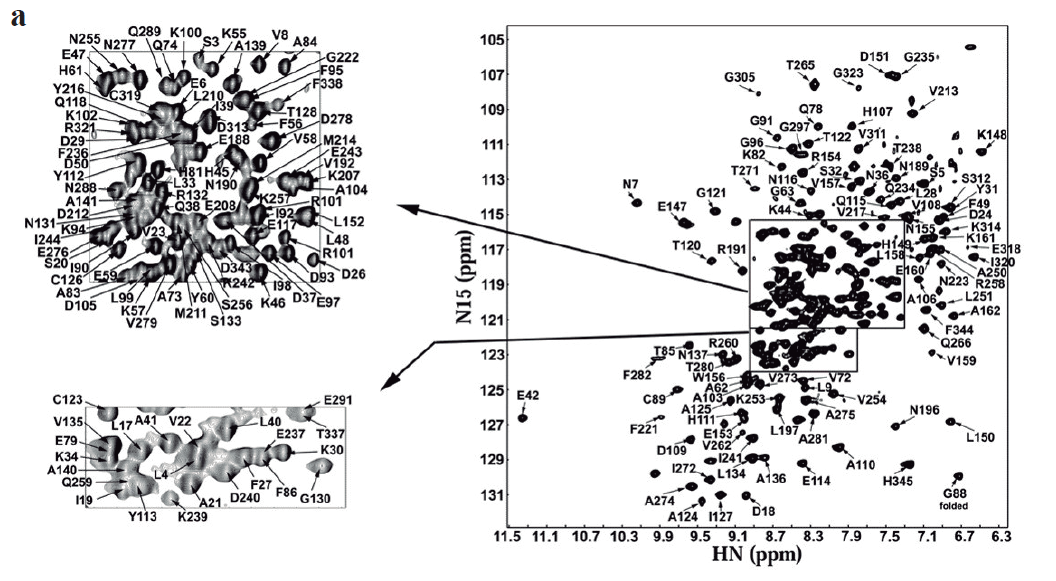


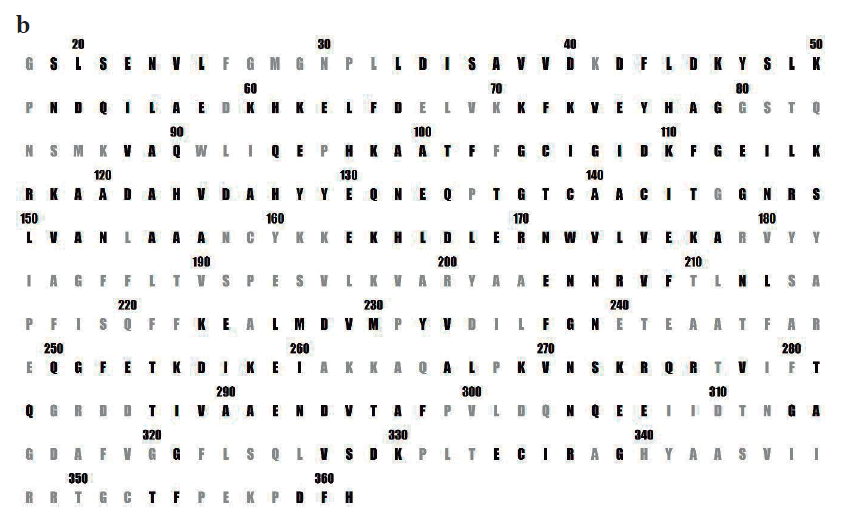


**Supplementary Figure S6**


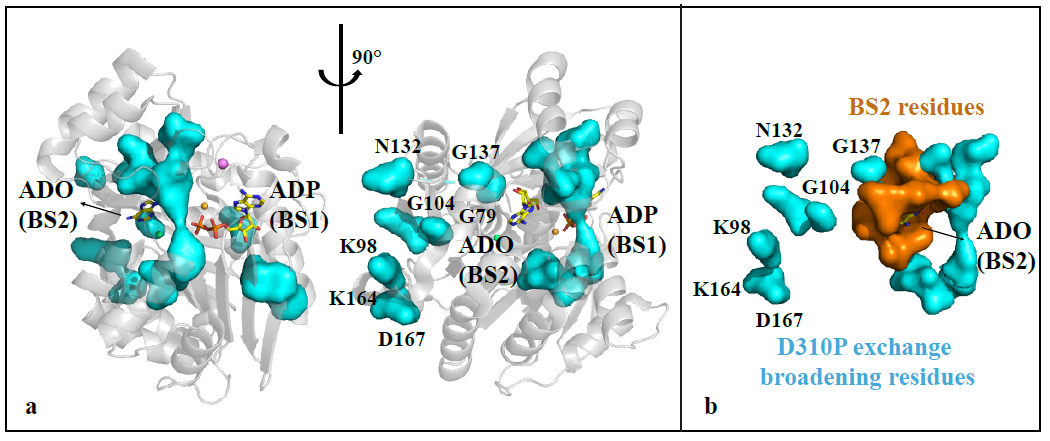


**Supplementary Table 1.** mADK kinetic parameters

|  | **^a^ADO** | |  | **^b^ATP** | | |  |
| --- | --- | --- | --- | --- | --- | --- | --- |
|  | **WT** | **D310P** | **D310A** | **WT** | | **D310P** | **D310A** |
| ***V*max**  **(µM ADP s^-1^ µg^-1^)** | 12.79(±1.20) | 9.26(±397) | 2.59(±0.27) | 7.84(±0.08) | 1.15(±0.06) | | 2.63(±0.06) |
| ***K*m (µM)** | 2.30(±0,38) | 12.81(±0,65) | 7.63(±1.39) | 3,53(±0,14) | | 33,03(±2,28) | 23.12(±1.81) |
| ***K*cat (s^-1^)** | 0.53 | 0.09 | 0.1 | 0.33 | | 0.05 | 0.1 |
| ***K*cat/ *K*m (M ^-1^ s^-1^)** | 2,30x10^5^ | 7.28x10^4^ | 1.31x10^4^ | 1.00x10^5^ | | 1.5x10^3^ | 4.32x10^3^ |

# a- kinetic constants obtained by substrate inhibition fitting model. b- kinetic constants obtained by fitting the Michaelis-Menten equation. Results are expressed as mean ± SD of six independent experiments

**Supplementary Table 2.** mADK kinetic parameters for the substrate competition assay.

| **^a^ATP kinetic constants** | | | | |
| --- | --- | --- | --- | --- |
| **ADO (µM)** | **5** | **10** | **20** | **40** |
| ***V*max**  **(µmol ADP s^-1^ µg^-1^)** | 7.03 (±0,05) | 10.25 (±0,10) | 16.17 (±0,30) | 12,49 (±0,11) |
| ***K*m (µM)** | 0.74(±0.06) | 1.82(±0.14) | 6.99(±0.73) | 6.64(±0.33) |
| **R square** | 0,9856 | 0,9799 | 0,9746 | 0,9822 |

# a- kinetic constants obtained by non-linear Michaelis-Menten fitting. The initial rate of reaction was obtained as a function of ATP and stepwise increases of ADO concentration. Measurements were obtained from 3 independent experiments. Results are expressed as mean ± SD of three independent experiments

**Supplementary Table 3.** Kinetic parameters of mADK for ATP in the presence and absence of potassium

|  | **KCl** | ***K*m (µM)** | ***V*max (µM s^-1^)** | ***K*cat (s^-1^)** | ***K*cat/ *K*m (M ^-1^ s^-1^)** |
| --- | --- | --- | --- | --- | --- |
| **^a^ATP** | **-** | 2.42 (±0.13) | 0.004 | 0.25 | 1.03x10^5^ |
|  | **+** | 2.10 (±0.12) | 0.007 | 0.46 | 2.19x10^5^ |

1. kinetic constants obtained by non-linear Michaelis-Menten fitting. Results are expressed as mean ± SD of six independent experiments.

**Supplementary References**

1. Mathews, II, M. D. Erion, S. E. Ealick, Structure of human adenosine kinase at 1.5 A resolution. *Biochemistry* **37**, 15607-15620 (1998).
